# Evaluation of Bilateral Vestibular Dysfunction in Iranian Adults and Elderlies by Electronystagmography and Video Head Impulse Test

**Published:** 2019-03

**Authors:** Sadegh Jafarzadeh, Mohammad Reza Golrokhian Sani, Nematollah Mokhtari Amirmajdi, Masoud Firouzi

**Affiliations:** 1 *Department of Audiology,* * School of Paramedical Sciences, Mashhad University of Medical Sciences, Mashhad, Iran.*; 2 *Department of * *Otolaryngology, Sina Hospital, Mashhad, Iran.*; 3 *Department of * *Otolaryngology, Mashhad University of Medical Sciences, Mashhad, Iran.*; 4 *Markazi Vestibular Assessment and Rehabilitation Center, Mashhad, Iran.*

**Keywords:** Aminoglycoside, Bilateral vestibular dysfunction, Caloric test, Ototoxicity, Video head impulse test

## Abstract

**Introduction:**

Vestibular abnormalities are common problems in the whole world, which can lead to bilateral vestibular dysfunction (BVD). That results in symptoms, such as vertigo, unsteadiness, falling, oscillopsia, and lower quality of life. The Objective of this study was to determine BVD in adults and elderlies with vertigo and unsteadiness.

**Materials and Methods:**

This study was conducted on 384 patients in two categories of adults (age range of 18-64 years) and elderlies (65 years old and above) through Electronystagmography (ENG), including caloric test and video head impulse test (vHIT). Patients called bilateral vestibular dysfunction when they have an abnormal bilateral weakness (summation of nystagmus response less than 20 for 4 stimulations and less than 12 for each ear) in caloric test and their vHIT has a gain lower than 0.6. The results of caloric tests were categorized into four groups, including normal, unilateral weakness, bilateral weakness, and central abnormalities.

**Results:**

The obtained results revealed that the frequency of BVD is higher than previously reported data in the medical literature. The frequency of BVD was 10.9% for the investigated patients (39.1% abnormal caloric, 12.5% abnormal vHIT, and 10.9% abnormal in both tests). The 38.5% of elderly patients had bilateral abnormal results in both tests.

**Conclusion:**

The results of this study showed BVD in some cases by caloric and vHIT tests. Elderlies showed more cases of BVD compared to adult patients.

## Introduction

Vestibular abnormalities are common problems in the whole world (1), which can lead to different complications, including bilateral vestibular dysfunction (BVD). Many conditions, such as ototoxicity, bilateral Meniere’ disease, meningitis, autoimmune diseases, labyrinthitis can lead to bilateral vestibular dysfunctions (2-5). The most common cause of BVD in the adult population is ototoxicity, which can occur with an aminoglycoside (2,3). The BVD is generally considered uncommon in vestibular abnormalities. The rate of BVD is reported within the range of 1.6-5%. The prevalence of vertigo is higher in the elderly population due to the aging process (6,7); therefore, more people may have BVD in advanced age.The symptoms of BVD include vertigo, unsteadiness (2), increased risk of falling down, oscillopsia, restricted physical activity leading to the lower quality of life (2,8-13). To the best knowledge of researchers, the rate of BVD is higher in the northeast of Iran. It may be due to the widespread prescription of aminoglycosides, especially Gentamicin. The objective of this study was to evaluate BVD in adults and elderlies with vertigo and unsteadiness.

## Materials and Methods

**Participants**

This study was conducted on 384 patients suffering from vestibular disorders with the symptoms of vertigo and unsteadiness, who underwent otologic and neurotologic examinations. The study population was selected from the severe cases of patients with vestibular vertigo in the northeast of Iran referring to Markazi Vestibular Assessment and Rehabilitation Center. The subjects were divided into two categories of adults (18-64 years old) and elderlies (65 years old and above). The inclusion criteria were vestibular vertigo and unsteadiness, absence of neurologic cause for vertigo, and normal magnetic resonance imaging (MRI) to rule out neurologic disorders if requested. The exclusion criteria were a conductive hearing loss or external and middle ear anomalies. The procedure explained in details for patients and informed consent was obtained from all the individuals included in the study.

**Procedure**

Vestibular assessments were conducted on patients who were asked not to take any prescribed medications for vertigo for at least two days before the tests. All the patients were evaluated by Electronystagmography (ENG). The ENG test (Hortmann, otometrics, Denmark) included spontaneous nystagmus, saccade, smooth pursuit, optokinetic, Gaze, head shake, modified Dix-hallpike maneuver, side- lying maneuver, roll, positional nystagmus, bi-thermal caloric, and visual fixation test. 

Saccade and Gaze tests were performed at the horizontal and vertical plane and smooth pursuit, optokinetic and head shake tests were conducted at the horizontal plane. Dix-hallpike maneuver, Side-lying maneuver, and Roll test were used for the diagnosis of benign paroxysmal positional vertigo (BPPV). Modified Dix- hallpike maneuver was performed with the examiner standing behind the patients. The patients turned their head to one side and then lied down. Head and neck were hyperextended and placed out of bed. The maneuver was performed for right and left sides. In some patients with neck or back problems, Side-lying maneuver was carried out instead of Dix- hallpike maneuver. Patients turned their head to one side and lied to the opposite direction. This maneuver also performed for both right and left sides. Roll test was employed for the evaluation of horizontal semicircular canal. 

Positional nystagmus was evaluated in several positions, including supine, supine head right, supine head left, whole body rotation to right and left sides. Patients also performed a task (counting numbers backward) during Positional nystagmus and Bi thermal caloric tests. The bithermal caloric test was carried out through warm and cold air irrigations for both ears at ±13ºC of body temperature. This test evaluated the very high-frequency omnidirectional range of horizontal semicircular canals in very low frequency. The otoscopic evaluation for each ear was performed once by an ENT specialist before patients’ referral and once by an audiologist in caloric position before irrigation. Additional cerumen was removed before irrigation. The patients also asked about the feeling of irrigation in the eardrum for 60 sec. Visual fixation performed for patients with measurable caloric nystagmus. 

Furthermore, some patients (n=64) were evaluated using the video head impulse test (vHIT) (ICS impulse 250 Hz, Otometrics, Denmark). In the vHIT test, patients were sitting and examiner standing behind them. Patients stared to a target in their front. For the evaluation of horizontal canals, examiner suddenly rotated the head in a horizontal plane. For vertical canals, the head was turned to one side first and then it was moved according to an appropriate plane. Patients were not aware of the direction of movement so they would not anticipate the directions of head movement. The VOR gains were evaluated for each canal. In addition, any catch-up saccade during and after head impulse was considered as abnormal. 

The BVD was diagnosed by no spontaneous and positional nystagmus and bilateral hypofunction in caloric and vHIT test (including overt nystagmus). Bilateral weakness in the caloric test was defined as the summation of SPV degree of nystagmus response less than 20 for 4 stimulations and less than 12 for each ear for warm and cold irrigations. Bilateral hypofunction in vHIT was defined as vistibulo ocular gain (VOR gain) lower than 0.6 in all 6 semicircular canals. Patients also asked about their aminoglycoside usage in the past several years.

**Data analysis**

Data were analyzed using SPSS software (version 19) and descriptive analysis was reported for Bilateral Weakness (BW) and BVD in percentage. 

## Results

A total of 384 patients were evaluated for vestibular abnormalities (Table.1). The majority of patients in the adult group were middle-aged.

**Table 1:** Age and sex of participants

	**number**	**Minimum age**	**Maximum age**	**Mean age (Standard deviation)**	**Sex (female)**
All patients	384	19	88	50.6 (13.8)	260 (67.7%)
Adults	320 (83.3%)	19	64	46.4 (10.5)	224 (70.0%)
Elderlies	64 (16.7%)	65	88	72.6 (5.9)	36 (56.3%)

*Caloric test*

The results of ENG and caloric test were categorized into four groups of 1) normal, 2) unilateral weakness, 3) bilateral weakness, and 4) Central abnormalities. Table 2 shows the results of ENG and caloric tests in these groups. The central abnormality diagnosed by abnormal ocular motor functions, vertical (downbeat) nystagmus in the positional test, very elevated SPV degree nystagmus in the caloric test, and inability at Visual fixation.

**Table 2:** Results of ENG and caloric tests in two groups

	**Normal**	**Unilateral weakness**	**Bilateral weakness**	**Central abnormality**
All patients	107 (27.9%)	118 (30.7%)	150 (39.1%)	9 (2.3%)
Adults	93 (29.1%)	98 (30.6%)	120 (37.5%)	9 (2.8%)
Elderlies	14 (21.9%)	20 (31.3%)	30 (46.9%)	0 (0%)

In a caloric test, 39.1% of our patients have the bilateral weakness. The difference between adults and elderlies is only 5%.

*vHIT test*

Some of the patients showed abnormal results in the vHIT test. Table 3 shows the results of vHIT in adult and elderly patients.

**Table 3:** Results of vHIT in the two groups of patients

	**n**	**Normal**	**Unilateral**	**Bilateral**
All patients	64	39 (60.9%)	17 (26.6%)	8 (12.5%)
Adults	51	34 (66.7%)	14 (27.5%)	3 (5.9%)
Elderlies	13	5 (38.5%)	3 (23.0%)	5 (38.5%)

*Evaluation of BVD in patients*

The BVD was defined as bilateral abnormal results in both caloric and v-HIT test; therefore, a total of 7 out of 64 (10.9%) patients including 2 (3.9%) adults and 5 (38.5%) elderlies had BVD. The age of patients also had a significant difference in patients with BVD and without BVD in t-test (P=0.002).Patients were asked about their aminoglycoside usage. Many patients were uncertain about their usage, especially in the elderly group. However, the higher percentage of BVD in elderlies may not exclusively be related to the aging process. It is possible that the use of more medication including ototoxic medication in elderlies could be a factor.

## Discussion

The result of the current study showed a relatively high ratio of BVD in the investigated patients. A large number of the investigated patients had bilateral weakness in the caloric test but fewer patients showed bilateral hypofunction in vHIT test. Accordingly, caloric test stimulates the vestibular system in very low frequencies and vHIT shows the function of the higher frequency region. This results may be related to the initial damages of the vestibular system in ototoxicity (14). The damage of vestibular function in ototoxicity starts at lower frequencies. 

The BVD is considered as a rare condition in patients with the vestibular disorder (15). It is not a common disorder in the developed countries because they have limited aminoglycosides to the special patients (15). In 1989, McGath diagnosed BVD by using ENG in only 1.6% of 5499 patients with the vestibular disorder (16). Patients with different diseases are treated with aminoglycoside, especially Gentamicin. These treatments cause ototoxicity for the vestibular system that counts for the main cause for developing BVD (2,3,16). 

Unfortunately, we could not document aminoglycoside usage in our patients because some of the patients, especially in the elderly group, do not correctly remember their usage in the past several years. On the other hand, vestibular evaluations and case history of most diagnosed patients did not comply with bilateral Meniere’ disease, Meningitis, or Labyrinthitis. Moreover, the aging process could not justify the high number of BVD in advanced age. It may be related to the widespread prescription of aminoglycosides in the hospitals and outpatients’ clinics. Currently, physicians could replace aminoglycoside (e.g., Gentamicin), which is available in most pharmacies, with other medications (e.g., the third generation of cephalosporin). The restriction for prescribing aminoglycoside could prevent the negative impacts of BVD, such as vertigo, oscillopsia, imbalance, falling down, especially in elderlies that could dramatically decrease the patient’s quality of life (8,12,13,17,18).

## Conclusion

The results of this study showed BVD in some cases by caloric and vHIT tests. Elderlies showed more cases of BVD than adult patients.
